# Mass-univariate analysis of scalp ERPs reveals large effects of gaze fixation location during face processing that only weakly interact with face emotional expression

**DOI:** 10.1038/s41598-023-44355-5

**Published:** 2023-10-09

**Authors:** Roxane J. Itier, Amie J. Durston

**Affiliations:** Department of Psychology, University of Waterloo, 200 University Avenue West, Waterloo, ON N2L 3G1 Canada

**Keywords:** Perception, Neurophysiology

## Abstract

Decoding others’ facial expressions is critical for social functioning. To clarify the neural correlates of expression perception depending on where we look on the face, three combined gaze-contingent ERP experiments were analyzed using robust mass-univariate statistics. Regardless of task, fixation location impacted face processing from 50 to 350 ms, maximally around 120 ms, reflecting retinotopic mapping around C2 and P1 components. Fixation location also impacted majorly the N170-P2 interval while weak effects were seen at the face-sensitive N170 peak. Results question the widespread assumption that faces are processed holistically into an indecomposable perceptual whole around the N170. Rather, face processing is a complex and view-dependent process that continues well beyond the N170. Expression and fixation location interacted weakly during the P1-N170 interval, supporting a role for the mouth and left eye in fearful and happy expression decoding. Expression effects were weakest at the N170 peak but strongest around P2, especially for fear, reflecting task-independent affective processing. Results suggest N170 reflects a transition between processes rather than the maximum of a holistic face processing stage. Focus on this peak should be replaced by data-driven analyses of the epoch using robust statistics to fully unravel the early visual processing of faces and their affective content.

## Introduction

Facial expressions (of emotions) convey a wealth of information which accurate temporal processing is necessary for appropriate social interactions. Decades of research have investigated the temporal dynamics of this process using event related potentials (ERPs), with an emphasis on early visual ERPs. Here, we focus on fearful and happy expressions processing during the first 350 ms of vision.

P1 (~ 80–120 ms post-face onset) is sensitive to attentional modulations^1–3^ and to stimulus low-level characteristics including luminance, contrast, spatial frequencies^4^ and its increases for fearful compared to neutral expressions^5–7^ are attributed to attention capture triggered by these stimulus differences^8–11^ (e.g. larger white sclera in fearful eyes). The N170 component (~ 130–200 ms) represents the integration of facial features into a holistic face percept^4,12^ and its increases by fearful expressions^5,13–16^ are interpreted as reflecting sensitivity to the change in face configuration elicited by the expression (the wide-open eyes with elevated eyebrows, the round shaped mouth etc.). Some have suggested that N170 increases to fearful faces might reflect affective processing indexed by the early posterior negativity (EPN, ~ 150–350 ms) that follows N170 at the same electrodes. Indeed, the EPN is typically more negative for fearful than neutral expressions^14,15,17–21^ and could start during, and overlap with, the face-related activity indexed by the N170^18,19,22,23^. However, many other studies have failed to report modulations of these components by expression^8,10,24,25^ and the reasons for these inconsistencies are still unclear. Two main factors are considered here: the location of participants’ gaze on the face and the statistics used in most face ERP work.

The position of participant’s gaze on the face is an important yet rarely controlled factor. In gaze-contingent ERP studies, faces are presented only if participants fixate on the fixation cross for a certain duration assessed by eye-tracking. Face presentation is offset such that a given feature is located at fixation, ensuring neural activity recording to the face when that feature is foveated. These studies have reported larger N170s when fixation is on an eye compared to the nose or mouth^26–29^, an effect unrelated to foveal contrast or pixel intensity differences^29^. These findings are noteworthy given the holistic processing theory according to which faces are processed as a gestalt where features are perceptually “glued” into an undecomposable whole during the N170^30–32^. Such a holistic process predicts that gaze position is irrelevant during face configural encoding and is thus put into question by these N170 modulations with fixation location^33–35^. These findings are relevant for facial expressions given that free-viewing eye tracking paradigms suggest spontaneous saccades are made towards the eye region when viewing fearful faces but towards the mouth when viewing happy expressions^36,37^, even for face presentations as short as 150 ms^37,38^. It is thus possible that during classic ERP studies, small eye movements put gaze fixation on different features, contributing to the modulations of early ERPs with facial expressions.

Indeed, certain facial features convey information that is characteristic of specific expressions and contribute to their neural processing. For instance, the larger N170 and EPN for fearful compared to neutral faces disappear when the eyes are covered^39^ and the salience of a smile given by teeth brightness can be processed as early as 90 ms^40^. Response classification techniques such as Bubbles^41^ suggest the P1-N170 interval reflects the featural integration process which ends at the N170 peak when the feature diagnostic of a given expression is encoded (eyes/mouth for fearful/happy expressions respectively) and this process is task-dependent^42^. In real life, however, we generally see whole faces as opposed to face apertures/bubbles, which we explore with fixations^43,44^. Understanding whether fixating on a particular feature modulates differently the early ERP components recorded to a given expression will deepen our understanding of expression processing as it happens in daily life and may elucidate the inconsistencies in the field. Importantly, no study has systematically investigated the effects of face fixation location on the entire neural time course, including the N170 but also the P1, the P2 and the EPN. As gaze-contingent paradigms present faces in slightly different locations to put features at the fovea, changes in retinotopic location of the face and parafoveal features likely impact neural activity. Few studies have investigated retinotopic mapping on the early visual ERP components but they used checkerboards, did not employ eye trackers and focused on peaks as opposed to the whole epoch^45–48^. Whether fixation location impacts visual components in a retinotopic way for complex stimuli like faces is unknown.

As far as we know, only three gaze-contingent ERP experiments have looked at the neural processing of whole faces comparing fearful, happy, and neutral expressions while fixation was enforced on the four main features using eye-tracking^23,49^. These studies included a gender discrimination task^49^, an emotion discrimination task and an oddball task with response to flowers^23^. Although some expression effects were consistent across tasks and fixation locations, different interactions between emotion and fixation location were also seen depending on the task, especially across N170 and EPN. These inconsistencies might be related to experimental designs or to the type of analyses performed. Indeed, these studies^23,49^ used a classic analysis approach where, in addition to literature-based peaks, some effects were investigated based on visual inspection of grand-averages and many follow-up analyses were performed, which inflates Type I errors^50^. Some of the interactions between emotion and fixation location may thus be false positives. Furthermore, effects exist between ERP peaks^42,51–56^, which is highlighted when using mass univariate analysis (MUA)^53,57–59^. MUA can reduce Type II errors as it takes a data-driven approach where every time point at every electrode is analyzed. MUA also reduces Type I errors by using stringent controls for multiple comparisons, allowing results across the whole epoch and scalp without needing multiple analyses for each region of interest^50,60–62^.

**Figure 1:**
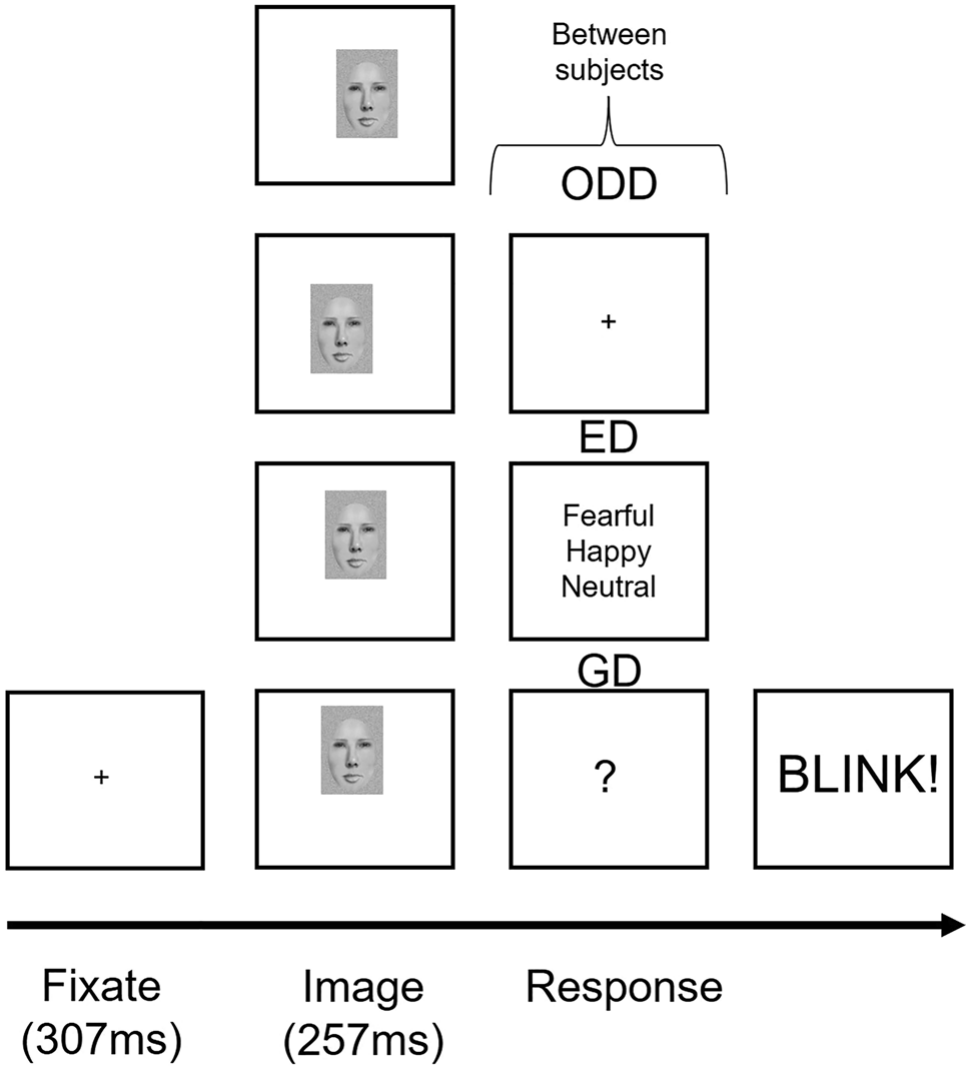
Schematic of the mixed model design and procedure. The three tasks used were between-subject factors. In the gender discrimination (GD) task, the response screen was a black question mark which prompted participants to differentiate the gender of the face using two buttons on a game controller. In the emotion discrimination (ED) task, participants selected the emotion of the face from a list on the screen using a mouse. In the oddball (ODD) task, participants pressed the spacebar on a keyboard when an infrequent flower stimulus was presented. A fixation cross was presented in lieu of the response screen for 747 ms on face trials and until a response on flower trials. The within-subject factors were Facial Expressions (fearful, happy, neutral) and Fixation Locations (left eye, right eye, nose, mouth). A gaze-contingent procedure was used wherein the successful fixation on the centered fixation cross for 307 ms (assessed online by an eye tracker) triggered the presentation of faces on the monitor. On every trial, faces were presented offset in such a way that the desired feature replaced the fixation cross at the fovea. Note that the face example is not a real photograph but was made in-lab using FACES™ 4.0 (IQBiometrix Inc) due to copyright issues, only for example purposes. The actual faces used during the study were from the NimStim database (Development of the MacBrain Face Stimulus Set was overseen by Nim Tottenham and supported by the John D. and Catherine T. MacArthur Foundation Research Network on Early Experience and Brain Development. Please contact Nim Tottenham at tott0006@tc.umn.edu for more information concerning the stimulus set). The models used in the present study were models # 2, 3, 6, 8, 20, 24, 33, 34).

The goal of the present study was to better our understanding of how feature fixation influences the early neural processing of emotional expressions when the whole face is presented, as would happen in everyday life. Given the uniqueness of their gaze-contingent approach and the use of identical stimuli, we combined the data from the three experiments^23,49^ and performed MUA, allowing for a larger sample size and increased power. Facial Expressions and Fixation Locations were within-subject factors and Task was a between-subject factor (Fig. 1). We expected that task would not significantly interact with facial expression^57,58^, an important result to replicate given the still largely inconsistent literature^25,63^. However, Task should interact with feature integration^42^ and thus with fixation location.

Enforcing fixation on specific face regions changed face position within the visual field so retinotopic effects were anticipated on early components. We expected replicating the largest P1 for mouth fixation^23,49^ and ipsilateral eye fixations^59^, due to the face situated mainly in the upper visual field or contralateral hemifield, respectively. These early effects would align with retinotopic mapping results^45^. We also expected to replicate the largest N170 for eye fixations^26–29^, argued to be high-level^28^. However, whether fixation effects could be seen after the N170 was unclear. P2 can be affected by stimulation in the upper visual field^45^ but based on the original results^23,49^, fixation effects were not expected past 200 ms, a finding that could be unique to faces. Indeed, the face-sensitive N170 is most likely coming from the inferior occipital gyrus and fusiform gyrus^64,65^, ventral areas known to contain cells with large receptive fields little impacted by face position in the visual field^66^. In terms of facial expressions, we anticipated increased N170 for fearful faces^25,67^, but also maximum “fear effect” around the P2 recently reported^57^, and possibly around P1 and EPN. Based on the literature reviewed above, a fixation by emotion interaction was predicted. Due to the importance of the eyes for fear processing, fearful expressions should differ from happy and neutral expressions when fixation was on one eye, and this could potentially be seen earlier than the N170. Additionally, we anticipated that fearful and happy expressions would differ from neutral ones when fixation was on the mouth, given the importance of the mouth for both expressions^68^, although this effect should be strongest for happy expressions due to the smile^40^.

## Results

**Figure 2:**
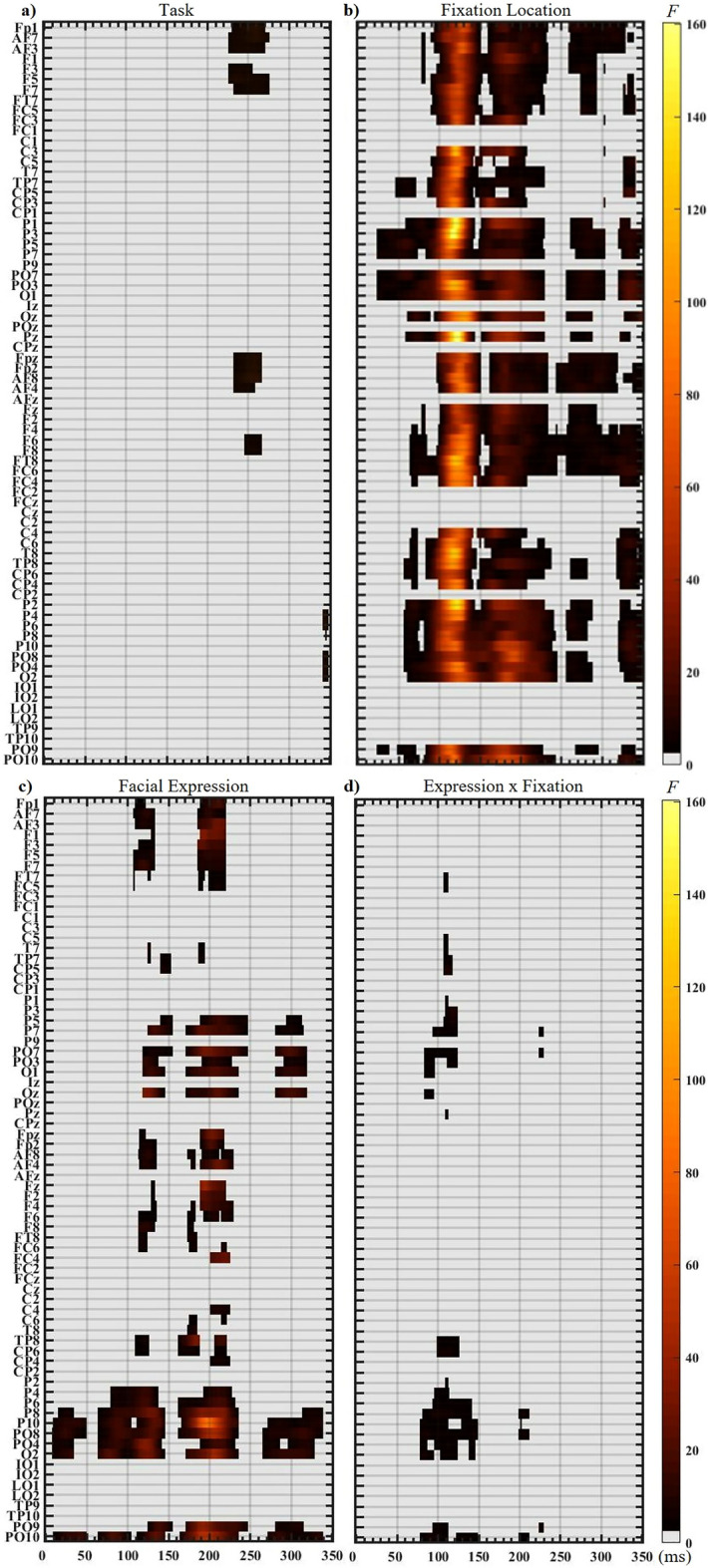
Main effects and interaction from the omnibus mixed model ANOVA (3 Tasks × 4 Fixation locations × 3 Facial Expressions), where Task was between-subjects and the other factors were within-subjects. The ANOVA was computed with *α* = 0.05. Results used the Threshold-Free Cluster Enhancement (TFCE) correction. (**a**) Task effect; (**b**) Fixation Location effect; (**c**) Facial Expression effect; (**d**) Facial Expression by Fixation Location interaction. Time is presented on the X-axis of each plot (from 0-352 ms after face onset), and electrodes are presented on the Y-axis. On the right side of each panel, a colour bar indicates the *F-*values strength. Task did not interact with any factor.

The mixed model ANOVA using a 3 Task × 4 Fixation Location × 3 Facial expression revealed significant main effects of Task, Fixation Location, Facial expression and an interaction between Fixation Location and Facial Expression (Fig. 2).

### Task effects

The between-subjects effect of Task was significant on two time-windows (Fig. 2a, Table 1). The first time-window lasted 226–278 ms at frontal sites and was due to less positive amplitude for the Oddball task compared to the other two tasks which did not differ. The second time window spanned 342–350 ms on right parietal and occipital sites, where more positive amplitude was seen for the gender discrimination (GD) task compared to the other two tasks. As expected, Task did not interact significantly with Facial Expressions. Unexpectedly, however, Task also did not interact with Fixation Location. Therefore, in the subsequent result sections we report only the within-subject analyses re-run without the Task factor to increase power. The results of these two analyses were extremely similar (compare Fig. 2 to the various effects reported in subsequent figures). These effects were also nearly identical between the TFCE (Fig. 2) and cluster-mass corrections (Sup. Fig. 1), indicating stable patterns.

**Table 1:** Task effects.

Timing	Location	Maximal F-value	Direction
226−278 ms	Fp1, AF7, AF3, F3, F5, F7, Fpz, Fp2, AF8, AF4, F6, F8,	259 ms @ AF8, *F*(2, 52) = 15.98, *P* = 0.036	(GD = ED) > ODD, *∆µV* = 1.59
342−350 ms	P4, P6, PO8, PO4, O2	344 ms @ P6, *F*(2,52) = 14.10, *P* = 0.038	GD > ED, *∆µV* = 2.39; GD > ODD, *∆µV* = 1.68

Timing and location of the activity obtained for the between-subject factor of Task in the omnibus mixed model mass-univariate ANOVA (see Fig. 2 for a visual representation). The maximum *F* value obtained for each temporal window is reported along with the direction of the effect and the amplitude difference (∆µV) between the three tasks.

### Fixation location effects (Fig. 3)

**Figure 3:**
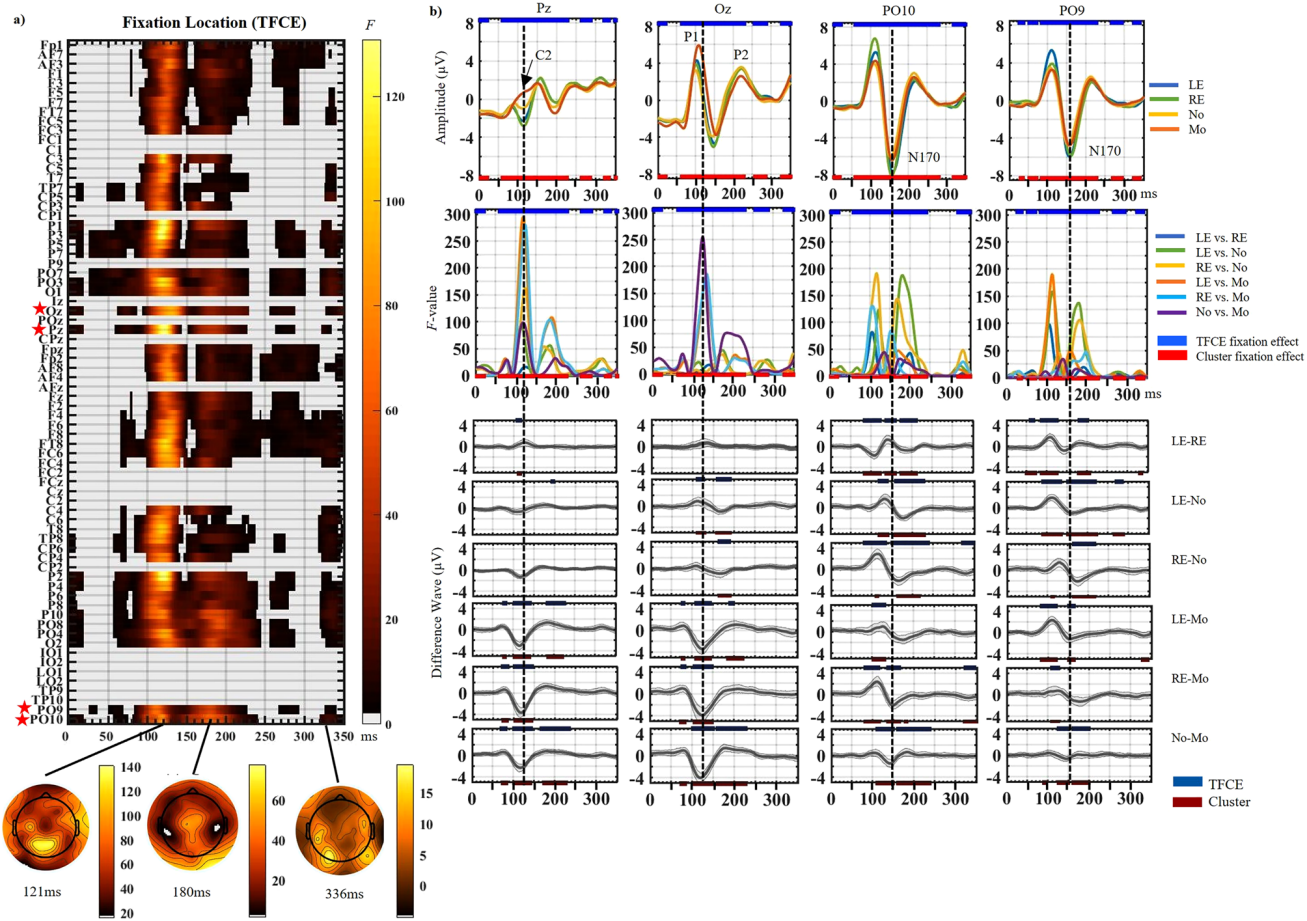
(a) Main effect of Fixation Location computed with α = 0.05 and TFCE correction. The raster plot depicts the significant *F*-values at every electrode (y axis) and time-point analyzed (0-352 ms post-face onset). *F*-values magnitude are according to the colored scale. Red stars highlight the electrodes that are zoomed on in (b): Pz, Oz, PO10, PO9. *LE* Left Eye fixation, *RE* Right eye fixation, *NO* Nose fixation, *MO* Mouth fixation. Feature position is from the observer’s viewpoint so the left eye means the eye on the left of the face image. The topographic maps of the unthresholded and uncorrected *F*-values are displayed at 121 ms, 180 ms and 336 ms when the *F*-value local maxima were registered for the effect of fixation after TFCE correction (see text and Table 2 for details). (b) Top row represents ERP plots showing the group averages for each fixation location at each of the 4 electrodes zoomed on. ERP waves were computed using inter-subject means and intra-subject 20% trimmed means. Middle row represents the *F*-values plots computed for each of the same 4 electrodes. Each line represents the time course of *F*-values obtained for each of the 6 contrasts (run at α = 0.008) comparing two fixation locations at a time. All contrasts show a peak of *F*-values between 100 and 150 ms more or less pronounced depending on the contrast and the electrode. Some electrodes also show a second peak between 150 and 200 ms. Note the large *F*-value peak at P10 for the left-eye-nose and right-eye-nose contrasts around 180 ms while weak *F* values are seen at the peak of the N170 component (dashed line). Blue lines at the top of each plots represent the time points at which the main effect of Fixation Location was significant at these electrodes using TFCE correction while the red lines at the bottom represent the time points at which the main effect of Fixation Location was significant using Cluster-mass correction (the two corrections elicited close to identical results). Bottom row: ERP difference waves for each fixation contrast at the same electrodes, computed using inter-subject means and intra-subject 20% trimmed means. Confidence intervals around the difference waves (i.e., Highest Density Interval; HDI) used α = 0.008 to align with the analyses. Dark blue lines at the top of the difference wave plots represent the points that were significant for a given contrast using TFCE correction while the dark red lines at the bottom of the same plots represent the time points that were significant for that contrast using Cluster-mass correction.

The main effect of Fixation Location was very strong, with many *F*-values up to 130. This effect was extant both spatially and temporally, seen across the scalp until 350 ms, with two clear “bands” of significance (Fig. 2b; Fig. 3a and Sup. Fig. 2 with different corrections; Table 2).

**Table 2:** Fixation location effects.

Timing	Location	Maximal F-value	Direction
0–19 ms	TP7, CP5, P1, P3, P5, P7, PO3, O1, Oz, Pz, TP8, CP6, P2, P4, P6, P8, P10, PO8, PO4, O2, PO10	All *F* < 11	
27–169 ms	Fp1, AF7, AF3, F1, F3, F5, F7, FT7, FC5, FC3, C3, C5, T7, TP7, CP5, CP3, P1, P3, P5, P7, PO7, PO3, O1, Oz, Pz, Fpz, Fp2, AF8, AF4, Fz, F2, F4, F6, F8, FT8, FC6, FC4, C4, C6, T8, TP8, CP6, CP4, P2, P4, P6, P8, P10, PO8, PO4, O2, PO9, PO10	121ms @ Pz (*F*(3, 51) = 130.85, *P* = 0.001)	Parietal (Pz): positive amplitudes for Mouth fixation, and negative for other locations Occipital (Oz): amplitudes were larger for Mouth than other locations Parietal-occipital: largest P1 for ipsilateral eye fixation
154–245 ms	Fp1, AF7, AF3, F1, F3, F5, F7, FT7, FC5, FC3, C3, C5, T7, TP7, CP5, CP3, P1, P3, P5, P7, PO7, PO3, O1, Oz, Pz, Fpz, Fp2, AF8, AF4, Fz, F2, F4, F6, F8, FT8, FC6, FC4, C4, C6, T8, TP8, CP6, CP4, P2, P4, P6, P8, P10, PO8, PO4, O2, PO9, PO10	180ms @ P10 (*F*(3, 51) = 64.57, *P* = 0.001)	More negative amplitude for LE and RE fixations than nose and mouth fixations on the N170-P2 interval
247–352 ms	Fp1, AF7, AF3, F1, F3, F5, F7, FT7, FC5, P1, P3, P5, P7, PO7, PO3, O1, Oz, Pz, Fpz, Fp2, AF8, AF4, Fz, F2, F4, F6, F8, FT8, FC6, FC4, TP8, C4, C6, T8, TP8, CP6, CP4, P2, P4, P6, P8, P10, PO8, PO4, O2, PO9, PO10	PO3 @ 336ms, (*F*(3, 51) = 21.48, 336ms, *P* = 0.001)	

Timing and location of the activity obtained for the Fixation Location within-subject factor in the mass-univariate ANOVA (see Fig. 3). The maximum *F* values obtained for each temporal window is reported along with the direction of the effect. Timings are the outermost significant datapoints for each “band”. Many electrodes had shorter significance bands falling within the time windows reported. Also, many electrodes were significant continually, during the entire activity window. See main text and Supplementary Table1 for more details on effect direction and paired contrasts.

The first and strongest “band” lasted from 27-169 ms across the scalp (different timing depending on sites; Fig. 3a, Table 2). The maximal *F*-value across this window, and the peak of the Fixation location effect, was at 121 ms on Pz, driven by positive amplitudes for Mouth location and negative amplitudes for the other locations (Fig. 3b, Pz; Sup. Table 1). This effect resembled the C2 component which is sensitive to retinotopic mapping. At occipital sites, amplitudes were largest for Mouth fixation on P1 and P1-N170 interval (Fig. 3b, Oz; see *F*-value plots). At parietal-occipital sites, however, the fixation effect was driven by largest P1 amplitudes for ipsilateral Eye fixation (Fig. 3b; PO10 & PO9; Sup. Table 1).

The next “band” lasted from 154 to 243 ms during the N170-P2 interval and including the P2. Here, the Fixation effect was driven by more negative amplitudes for Eye fixations compared to Nose (and to a lesser extent to Mouth) fixation at posterior lateral sites (Fig. 3b, PO9, PO10; see *F*-value plots). This result included the peak of the N170, but largest amplitude differences were seen *after* the peak (N170-P2 interval; see Fig. 3b vertical dashed lines for N170 peak). The local maximum for this “band” was at 180 ms on PO10, just before the P2 (Fig. 3b; Table 2), even though the N170 peak was clearly larger for Eye fixations compared to other fixations. The effect was also largest for the Left-eye fixation on both hemispheres (Fig. 3b PO9 & PO10 *F* value peaks). After 250 ms, *F* values were small (< 25) with no clear pattern of fixation effect emerging.

### Effect of facial expression (Fig. 4)

**Figure 4:**
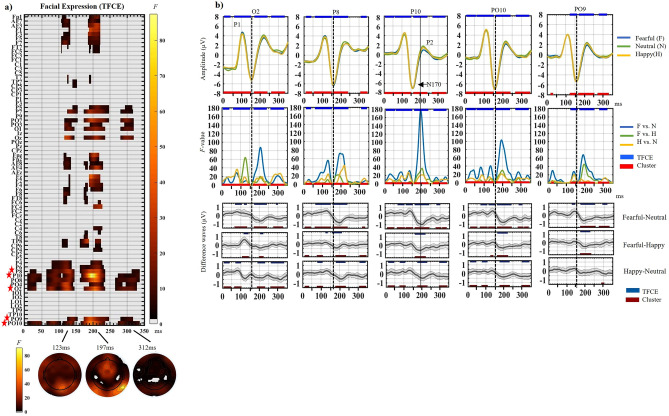
(a) Main effect of facial Expression computed with α = 0.05 and TFCE correction. The raster plots depict the significant *F*-values at every electrode (y axis) and time-point analyzed (0-352 ms post-face onset). *F*-values magnitude are according to the colored scale. Red stars highlight the electrodes that are zoomed on in (b). The topographic maps of the unthresholded and uncorrected *F*-values are displayed at 123 ms, 197 ms and 312 ms when the local *F*-value maxima were registered for the effect of expression after TFCE correction (see text for details). (b) Top row represents ERP plots showing the group averages for each facial expression at each of the 5 electrodes zoomed on (O2, P8, P10, PO10 and PO9). ERP waves were computed using inter-subject means and intra-subject 20% trimmed means. Middle row represents the *F*-value plots computed for each of the same 5 electrodes. Each line represents the time course of *F*-values obtained for each of the 3 contrasts comparing two expressions at a time (α = 0.016). Note the large *F*-value peak at P10 for the fearful-neutral contrast around the P2 (vertical plain line), and a similar, albeit smaller, peak at that timing at the other electrodes. In contrast, the weakest *F* values are seen at the peak of the N170 component (dashed lines). Blue lines at the top of each plot represent the time points at which the main effect of Expression was significant at these electrodes using TFCE correction while the red lines at the bottom represent the time points at which the main effect of Expression was significant using Cluster-mass correction (the two corrections elicited extremely similar results, see Sup. Fig. 2 for cluster mass results). Bottom row: ERP difference waves for each contrast at the same electrodes, computed using inter-subject means and intra-subject 20% trimmed means. Confidence intervals around the difference waves (i.e., Highest Density Interval; HDI) used α = 0.016 to align with the analyses. Dark blue lines at the top of the difference wave plots represent the points that were significant for a given contrast using TFCE correction while the dark red lines at the bottom of the same plots represent the time points that were significant for that contrast using Cluster-mass correction.

A main effect of Facial Expression was found (Fig. 4a, left panel), regardless of the correction used (Sup. Fig. 2 for the cluster-mass correction). This strong effect (*F* up to 87) was seen until 350 ms with four main time-windows (Fig. 4a, Table 3). The first window was brief (7–50 ms), with low *F*-values (< 16), and only on right parietal and occipital sites.

**Table 3:** Facial expression effects.

	Timing	Location	Maximal F-value	Direction
Main effect of facial expression	7–50 ms	PO10, O2, PO4, PO8, P10, P8	N/A (*F* < 16)	N/A
	65–155 ms	PO10, O2, PO4, PO8, P10, P8, P4, P6, Oz, PO9, O1, PO3, PO7, P7, P5, Fp1, AF7, AF3, F1, F3, F5, F7, FT7, FC5, Fpz, Fp2, AF8, AF4, Fz, F2, F4, F6, F8, FT8, FC6, CP6, TP8, CP5, TP7, T7	123 ms @ PO4, (*F*(2, 52) = 34.16, *P* = 0.001)	–
	161–247 ms	PO10, O2, PO4, PO8, P10, P8, P4, P6, PO9, Oz, PO9, O1, PO3, PO7, P7, P5, CP6, TP8, T8, C6, CP4, C4, TP7, T7, Fp1, AF7, AF3, F1, F3, F5, F7, FT7, FC5, Fpz, Fp2, AF8, AF4, Fz, F2, F4, F6, F8, FT8, FC6, FC4	197 ms @ PO10 (*F*(2, 52) = 87.46, *P* = 0.001) Also peak Facial Expression effect	–
	265–337 ms	PO10, O2, PO4, PO8, P10, P8, Oz, PO9, O1, PO3, PO7, P7, P5	300 ms @ PO7 (F(2, 52) = 19.15, *P* = 0.004)	–
Fear vs. neutral	72–146 ms	PO10, O2, PO4, PO8, P10, P8, P6, P4, PO9 P7, PO7	130 ms @ P8 (F(1, 53) = 56.10, *P* = 0.001,	∆µV = 0.59 (fear > neutral), [0.25; 0.89] 98.4% CI
	163–245 ms	PO10, O2, PO4, PO8, P10, P8, P6, P4, PO9 TP8, CP6, F6, F4, F2, Fz, AF4, AF8, Fp2, Fpz, Oz, O1, PO3, PO7, P7, P5, Fp1, AF7, AF3, F1, F3, F5, F7	198 ms @ P10 (F(1, 53) = 178.08, *P* = 0.001,	∆µV = − 0.96 (fear < neutral), [− 0.70; − 1.22] 98.4% CI)
	286–322 ms	PO10, PO9, P10, PO8, P7, PO7,	298 ms @ PO7 (F(1, 53) = 34.39, *P* = 0.016,	∆µV = − 0.46 (fear < neutral), [− 0.14; − 0.73] 98.4% CI)
Fear vs. happy	112–138 ms	AF7, F5, F7, P4, P6, P8, PO8, PO4, O2	123 ms @ PO4 (F(1, 53) = 68.17, *P* = 0.01,	∆µV = 0.65 (fear > happy), [0.34; 0.96] 98.4% CI)
	161–222 ms,	PO10, PO9, O2, PO8, P10, P8, P6, CP6, TP8, Oz, O1, PO7, P7,	184 ms @ TP8 (F(1, 53) = 51.19, *P* = 0.013,	∆µ = − 0.38 (fear < happy), [− 0.02; − 0.73] 98.4% CI)
Happy vs. neutral	10–39 ms	PO10, O2, PO4, PO8, P10	23 ms @ PO8 (F(1, 53) = 23.04, *P* = 0.002	∆µV = 0.40 (happy > neutral), [− 0.00; 0.60] 98.4% CI)
	73–103 ms	PO10, O2, PO4, PO8, P10, P8, P6, P4	82 ms @ O2 (F(1, 53) = 36.74, *P* = 0.001	∆µV = 0.49 (happy > neutral), [0.22; 0.82] 98.4% CI)
	179–227 ms	PO10, O2, PO4, PO8, P10, P8, P6, P4, CP6, TP8, Fp1, AF7	217 ms @ P8 (F(1, 53) = 44.21, *P* = 0.001	∆µV = − 0.49 (happy < neutral), [− 0.19; − 0.72] 98.4% CI)
	268–337 ms	PO10, O2, PO4, PO8, P10, P8, P6, P4, Oz, O1, PO3, PO7,	305 ms @ Oz (F(1, 53) = 29.57, *P* = 0.001	∆µV = − 0.41 (happy < neutral), [− 0.06; − 0.71] 98.4% CI)

Timing and location of the activity obtained for the main effect of Facial Expressions in the repeated measure mass-univariate ANOVA (see Fig. 4a). Results for the contrasts comparing two expressions at a time are also reported (see Sup. Fig. 4 for paired contrasts). The maximum *F* values obtained for each temporal window is reported along with the direction of the effect when applicable, and the amplitude difference (∆µV) between the three facial expressions.

The second window spanned 65–155 ms (P1 peak and P1-N170 interval) on right posterior sites, with restricted timing on a majority of other electrodes. Follow-up contrasts (Sup. Fig. 4, Table 3) revealed more positive amplitude for Fearful than Neutral expressions (72–146 ms, Fig. 4b difference wave plots), more positive amplitudes for Fearful than Happy expressions (112–138 ms), and more positive amplitudes for Happy than Neutral faces (73–103 ms, upstroke of the P1, see O2 on Fig. 4b).

The third window was seen from 161 to 247 ms over bilateral parietal/occipital electrodes (with shorter durations at other electrodes) with maximal *F*-values on right sites. The peak of this window, and of the main effect, was on P10 at 197 ms, representing lower amplitudes during the N170-P2 interval and P2 peak for Fearful than Neutral expressions (163-245 ms; Fig. 4b *F*-value plot, plain vertical line; Table 3, Sup. Fig. 4). Amplitudes were also lower for Fearful than Happy expressions (161–222 ms) and lower (although weakly) for Happy than Neutral expressions (179–227 ms, Table 3, Fig. 4b). Importantly, at the electrodes where the N170 is typically measured, the peak of the N170 (~ 150 ms) corresponded with some of the lowest *F*-values (vertical dashed lines on Fig. 4b).

The final window spanned 265–337 ms on parietal/occipital electrodes and included lower *F*-values (< 20), with a local peak at 300 ms on PO7 (Table 3, Fig. 4a). This timing corresponded to the EPN component with more negative amplitudes for Fearful than Neutral faces (286–322 ms) and for Happy than Neutral faces (268–337 ms).

### Fixation location by expression interaction (Fig. 5)

**Figure 5:**
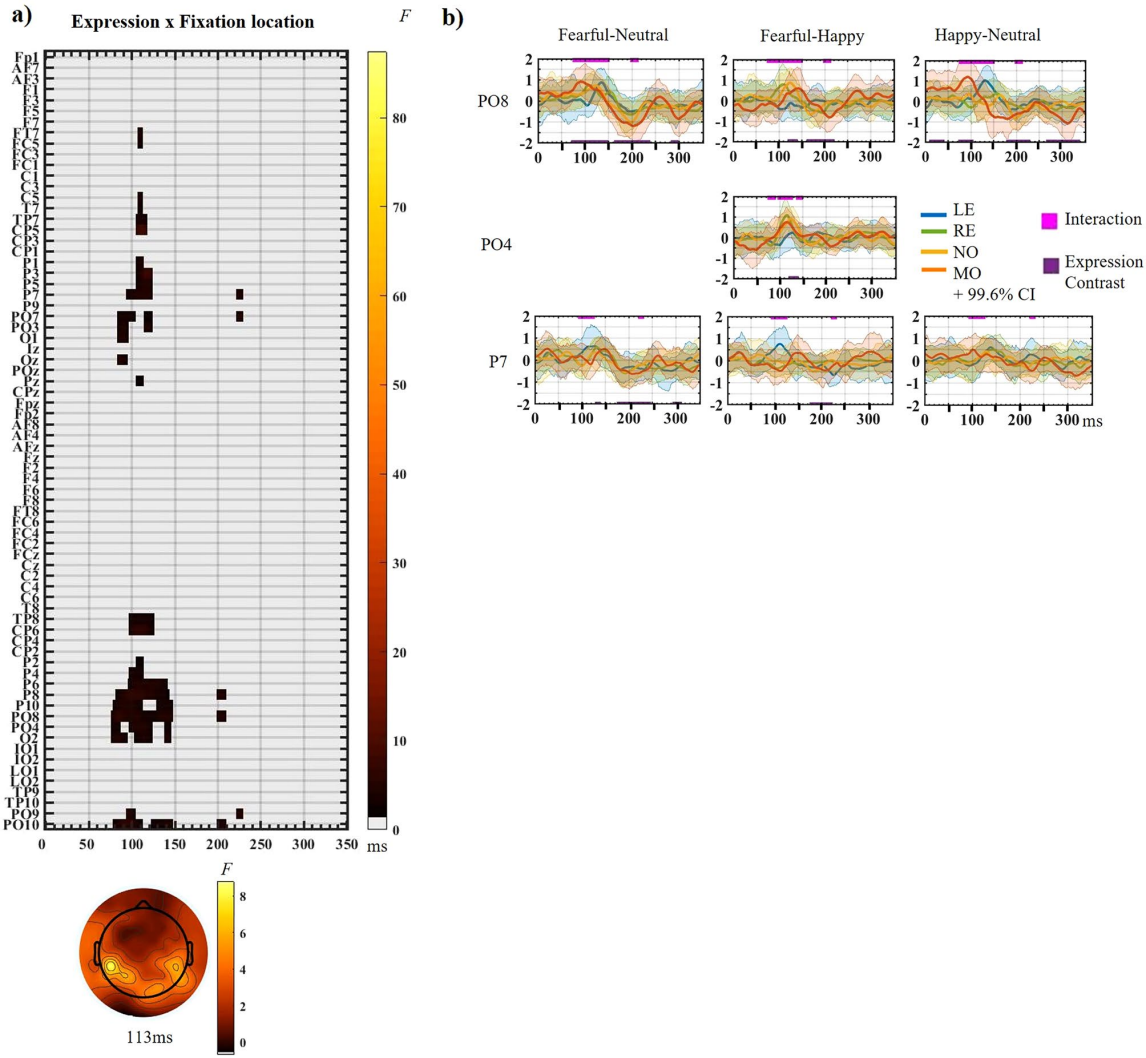
Interaction between Expression and Fixation Location computed with α = 0.05 and TFCE correction applied (see Sup. Fig. 2 for the interaction with cluster-mass correction). (a) The raster plots depict the significant *F*-values at every electrode (y axis) and time-point analyzed (0–352 ms post-face onset). *F*-value magnitude is according to the colored scale. The topographic map of the unthresholded and uncorrected *F*-values is displayed at 113 ms, the local *F*-value maximum for the interaction registered after TFCE correction (see text for details). (b) Difference wave plots displaying each expression contrast at the four fixation locations (*LE* Left eye fixation, *RE* Right Eye fixation, *NO* Nose fixation, *MO* mouth fixation). From left to right: Fearful-Neutral, Fearful-Happy and Happy-Neutral contrasts, displayed at PO8 electrode (top row), PO4 electrode (middle row) and P7 electrode (bottom row). ERPs and difference waves were computed using inter-subject means and intra-subject 20% trimmed means. Confidence intervals around the difference waves (i.e., Highest Density Interval; HDI) used α = 0.004 to align with the analyses. Pink lines at the top of the difference wave plots represent the points that were significant for the Expression by Fixation Location interaction while the dark purple lines at the bottom of the same plots represent the time points that were significant for the Expression contrast focused on (both using TFCE correction).

A significant, albeit weak, interaction between facial expression and fixation location was found from 76 to 148 ms (*F* < 10; Fig. 5a). This effect was mainly on right parietal and occipital electrodes and was driven by temporal differences between fixation locations (Fig. 5b, PO8). Before 100 ms (P1 upstroke), the Fearful-Neutral and Happy-Neutral contrasts were largest at Mouth fixation (dark orange lines on Fig. 5b). From ~ 100 to 148 ms (P1-N170 interval), the two contrasts differed maximally for Mouth (closer to P1) and Left eye (closer to N170; Fig. 5b PO8). The Fearful-Happy contrast (around 117 ms) was maximal for Right-eye fixation and smallest for Left-eye fixation (Fig. 5b PO4, PO8).

The interaction was also brief from 199 to 210 ms at P8, PO8 and PO10 (Fig. 5a), driven by largest expression differences for Mouth and Nose fixations compared to the Eye fixations. This was most prominent for Fearful-Neutral and Fearful-Happy contrasts (Fig. 5b PO8).

Lastly, the interaction was seen on left electrodes between 84 and 126 ms, driven by the Fearful-Happy and the Fearful-Neutral contrasts being largest for Left eye fixation around 120 ms (P1-N170 interval; Fig. 5b P7). A similar pattern was seen transiently between 222 and 230 ms for the Happy-Neutral contrast. The peak of the whole interaction was seen on CP5 at 113 ms (*F*(6, 48) = 8.76, *P* = 0.026), driven by largest Fearful-Neutral and Fearful-Happy differences at Left-eye fixation.

## Discussion

Decades of research on the neural processing of facial expressions have elicited inconsistent findings regarding their temporal dynamics^25^. Two important factors have been neglected: the position of participants’ gaze on the face and the type of statistical analyses used, most studies employing classic ERP analyses that inflate Type I and Type II statistical errors and contribute to the replication crisis in this field^50,60–62^. The present study took advantage of three unique gaze-contingent experiments where gaze fixation was carefully controlled and employed robust mass-univariate analyses (MUA) allowing the study of the entire time-course. We found strong and widespread effects of Fixation Location and Expression which interacted only weakly, while Task did not modulate any factor. We discuss the implication of these effects for face processing.

### Effects of fixation location

Where participants’ gaze was fixated strongly impacted neural activity across most of the epoch and electrodes, being strongest between 80 and 250 ms (Figs. 2b, 3a). Retinotopic effects were anticipated early, due to face position changes. When fixation was on the mouth, most of the face was in the upper visual field, while it was in the lower visual field for the other three fixation locations. When fixation was on one eye (e.g. left) most of the face was situated in the opposite (right) hemifield, processed by the contralateral (left) hemisphere, i.e. ipsilaterally to the fixated (left) eye. These anticipated upper/lower and left/right hemifield effects were found, replicating largest P1 for mouth fixation^23,49^ and ipsilateral eye fixations^59^. In addition, MUA revealed that the strongest effects of fixation location encompassed the equivalent of the C2 component and the P1-N170 interval.

The peak of the Fixation location effect was at 121 ms at Pz, also visible on the occipital P1 (see topography on Fig. 3a and ERPs on Fig. 3b), driven by most positive amplitudes for Mouth fixation, and negative amplitudes for the other fixations. This amplitude pattern resembles that of the C2 (also called P125/N135) reported at centro-parietal sites^45–47^, where C2 is positive for upper visual field and negative for lower visual field stimulations using checkerboards, maximally so across the vertical median^45^. Finding a C2 equivalent for face stimuli is novel and this early advantage for the upper visual field could be related to the behavioural upper visual field advantage in processing face gender^69^ and gaze direction^70^, although brain-behaviour studies are needed to investigate possible cascading effects of retinotopic position onto behavioural face processing asymmetries.

Largest P1s for ipsilateral eye fixation^23,26,28,49,71^ were found in every contrast (Fig. 3b) and the fixation effect emerged as early as 50 ms at left sites (Fig. 3a), driven by differences between the two eye fixations (Sup. Fig. 3a) during P1 upstroke. A similar effect was reported when only the face outline was present (no other feature than the fixated eyes) but not when the same eyes were presented in isolation^59^. Therefore, this early effect seems driven by the face contour and the face portion it delineates, which is processed contralaterally.

The change in fixation location affects the position of both the face and the parafoveal features. When fixation is on the left eye, the right eye is in the right hemifield at the same vertical level but when fixation is on the mouth, the right eye is in the upper right field. The varying position of the parafoveal features in the visual field changes their coding and integration into the face percept, which some have suggested occurs during the P1-N170 interval, culminating with the N170 peak when expression-diagnostic features have been encoded^42,51,56^. These studies presented face portions to participants through apertures (bubbles) of different sizes and spatial frequencies and used reverse correlation techniques to determine what visual information is used for the task at hand. Here, only whole faces were presented, which better corresponds to our daily experience where we move our eyes across the face^43^, hereby changing foveal and parafoveal input. Our early amplitude variations are in line with this feature integration process during P1-N170. However, our results also suggest that feature integration continues well past the N170 until 250 ms (Fig. 3a), in contrast to the original findings^23,49^.

Indeed, while the anticipated largest N170 for eye fixations^23,26–28,49^ was replicated, the second strongest effect of fixation location was seen during the N170-P2 interval. The N170 peak was a time point where small *F*-values were seen (Fig. 3b), only reflecting the beginning of the N170-P2 interval effect. This interval codes the ipsilateral eye^51^ and the number of parafoveal features^59^ while the P2 itself is maximally sensitive to the type of parafoveal feature^59^. Retinotopic stimulations have shown an upper visual field advantage around P2^45^, while largest differences were seen between eye and nose fixations, suggesting the effects during this N170-P2 interval are not retinotopic but possibly reflect face-specific coding processes. Furthermore, task demands did not interact with the fixation effect during any time point, suggesting the neural integration of facial features into a face representation is largely task-independent, in contrast to previous suggestion^42^. We propose that face feature coding involves feedback loops within the face processing network that are responsible for the fixation effects seen until 250 ms, an idea that needs further testing.

Thus, solely focusing on the N170, where small fixation effects were seen, results in missing the strongest effects of fixation location. Importantly, the widely accepted view that the N170 reflects holistic processing^4,33^ in the sense of a perceptual snapshot, is inaccurate. The perceptual construction of a face is dynamic, malleable, varies with fixation position and occurs across a longer time frame than marked by the N170. Rather, the N170 peak seems to mark an inflection point between two processes, the first reflecting the retinotopic mapping effects driven by face position in the visual field during which feature integration into a face percept begins, the second seemingly reflecting the continuation of feature coding and integration during the N170-P2 interval with an emphasis on the eyes. More gaze-contingent studies taking a whole epoch approach are needed to unravel precisely feature integration during the first 350 ms of vision.

### Effects of facial expression of emotion

The effect of facial expression was strong and seen throughout the epoch at many electrodes. Although bilateral, the effect was largest on right posterior sites, in agreement with a wealth of research^72^. Importantly, and as predicted, the between-subject factor of task did not interact with expression, replicating recent MUA studies using within-subjects tasks^57,58^. These results suggest, in contrast to recent claims, that the early (up to 350 ms) processing of facial expressions occurs regardless of the attentional and cognitive demands imposed by the tasks^25,73,74^.

Early activity surrounding the P1 and P1-N170 interval differentiated between the three facial expressions (Fig. 4), fearful faces eliciting larger amplitudes compared to happy and neutral faces (Fig. 4b, O2, P8). Previous peak studies reported similar fearful-neutral differences^5–7^, interpreted as attentional capture by low level stimulus variations such as spatial frequencies^11,75^, indicative of threat-related expressions^8–10^. Early amplitude modulations around the P1 have also been reported for happy faces, explained by the saliency and luminance of smiles^40^, possibly enhancing the overall processing of happy expressions^76^. Given the previously reported lack of early effects for happy faces with nose fixation^57^, it is likely that our early effects for happy faces are driven by the smile, a suggestion supported by our interaction results. Indeed, the earliest differences between the expressions were seen during the P1 upstroke with mouth fixation. Additionally, the strongest differences between fearful and the other expressions was during the P1-N170 interval with left eye fixation, during which features, especially the left eye, are encoded^42,51,56^. This interaction was small and will need replication but suggests that low-level local differences between features contribute to the early differences between expressions. As discussed earlier, and in contrast to earlier reports^42^, this featural decoding seems immune to task demands. Rather, feature integration varies (weakly) with facial expressions regardless of what task participants are engaged in.

The expression effect was strongest around the P2 component on right lateral parietal sites and was driven by most negative amplitudes for fearful faces, and more negative amplitudes for happy compared to neutral faces (Fig. 4). This effect is remarkably similar to a recent MUA^57^ where the expression effect was maximal around similar timing at the same electrode (P10). Aside from a very transient and weak interaction (199–210 ms), expression and feature integration were processed largely in parallel during the N170-P2 interval. Much research employing classical peak-based analyses has shown sensitivity of the N170 to fearful^25,67^ faces although many inconsistencies remain^24,25^. Critically, our results indicate that the N170 peak is the time point around which emotion effects are the weakest (Fig. 4b), explaining the inconsistent N170 expression effects in the literature which are simply the beginning of the effects seen during the N170-P2 interval. Therefore, focusing on the N170 peak, rather than the whole epoch, will result in missing the strongest emotion effects.

This strong expression effect around the P2 has been interpreted^57^ as the decoding of threat-specific affective content (valence and arousal) that are classically associated with the EPN. Some have suggested that N170-P2 interval represents an early EPN superimposed on the N170 component^18,19,22,57^. This timing coincides with amygdala discharges recorded intracranially between 150 and 200 ms in response to fearful faces^77–79^. Recent intracranial studies show amygdala responses to fearful faces as early as 75–80 ms after face onset, driven by low spatial frequencies and seen even when the faces were invisible to participants^80,81^. However, responses from the face sensitive fusiform gyrus only differentiated between fearful and neutral faces from 172 to 218 ms and not earlier^80^. Therefore, the emotion effect seen on scalp between 160 and 250 ms could reflect modulations of the cortical face processing network by amygdala projections, given the bidirectional connections between amygdala and fusiform gyrus^82^ (the amygdala itself cannot be recorded on the scalp). Accordingly, the weaker effects seen for happy and fearful faces during that interval would simply relate to the weaker involvement of the amygdala for other expressions than fearful ones^83,84^. The expression effects around and during the P2 peak are rarely investigated^25^ and if found, not discussed^85–87^. Although the expression effect around the P2 seems related to the affective content^88^, more work is needed to understand this effect and its relationships with expression intensity, valence and arousal.

In the present study we found a weak effect of expression during the classic EPN timing (265–337 ms), driven by differences between neutral and emotional expressions and clearly distinct from the N170-P2 modulations. While emotion modulations on the EPN are common, much inconsistency remains^14,15,17–19,21,25^. The present study showed EPN modulation by happy expressions, along with restricted modulation by fearful expressions, in contrast to a recent MUA^57^. Despite using the exact same tasks and faces as the present study, ruling out stimulus related differences, that study only included 24 participants (less than half of our sample), potentially highlighting individual differences or even type I errors, in these later effects which need replication.

## Conclusions

Most ERP studies continue to focus on peaks and on the N170. Using a data-driven mass-univariate analysis approach, we showed that, in contrast to the classic assumption that the N170 reflects a holistic process where features are glued into an indecomposable face percept, feature integration is a very dynamic and malleable process which starts early and varies with fixation location on the face until past 250 ms. Feature integration seems immune to task demands, strongest during the P1-N170 and N170-P2 intervals and is weak at the peak of the N170. We propose that this complex feature integration process involves complex feedback loops withing the face processing neural network, seen as processing waves at different intervals but similar electrodes. Facial features contribute early (P1-N170) but weakly to the decoding of the facial expressions, with a seemingly largest role of the mouth and left eye, while the affective content of faces is processed essentially *after* the N170, at which time effects are weakest, and mainly around the P2. This expression effect is immune to task demands, largely independent of gaze location and is strongest for fearful faces although clearly seen for happy faces. The sole focus on the N170 peak in face and facial expression research should be avoided. More studies using data-driven approaches, robust statistics, large sample sizes and gaze-contingent designs are needed to continue unravelling the early visual processing of faces and their affective content.

## Material and methods

### Participants

All participants provided informed written consent before the experiment. This project received ethic clearance from the University of Waterloo’s Research Ethics Board and complied with the Declaration of Helsinki. The original studies included a final sample size of 66 participants, 20 in the Gender Discrimination (GD) task^49^, 20 in the Emotion Discrimination (ED) task, and 26 in the oddball (ODD) task^23^. As MUA requires a large number of trials per condition for reliability due to bootstrapping methods^89^, 12 participants were rejected. The final sample included 54 participants: 19 in the ED task (10 males), 17 in the GD task (8 males) and 18 in the ODD task (7 males). All participants were between 18 and 25 years of age and within the normal range of anxiety (scores < 43 on the State-Trait Inventory for Cognitive and Somatic Anxiety; STICSA^90^).

### Stimuli and design

Eight identities (4 female, 4 male) from the NIMSTIM database^91^ each expressing fearful (F), happy (H), and neutral (N) expressions were edited to remove peripheral distractors (e.g., hair, ears) using elliptical masks and were converted to grayscale. Six flowers were edited with the same procedure for use in the oddball task. Root Mean Square (RMS) contrast and pixel intensity (PI) were also calculated for each fixation location, using a 1.4° region of interest around the fixation location centered in the middle of the feature. In this study, feature position is from the observer’s viewpoint, so the left eye means the eye on the left side and the right eye is the eye on the right side of the face image. The three emotional expressions did not significantly differ on mean PI or RMS contrast, although features did and an interaction between feature and expression was seen^23^. PI was lowest and RMS contrast highest, for eye regions compared to nose and mouth (Table 4). There were no differences between emotion expressions for either eye region. However, PI for mouth fixation was largest for happy expressions.

**Table 4:** Mean RMS contrast and pixel intensity (averaged across the 8 faces used) for each fixation location and emotional expression (calculated for the pre-defined areas of interest of 1.4° around the fixation location), as originally reported^23,49^.

	Mean RMS contrast (SE)					Mean pixel intensity (SE)				
	Full face	Left eye	Right eye	Mouth	Nose	Full face	Left eye	Right eye	Mouth	Nose
Fearful	0.33 (0.01)	0.14 (0.01)	0.14 (0.01)	0.13 (0.02)	0.12 (0.01)	0.58 (0.01)	0.43 (0.02)	0.44 (0.04)	0.49 (0.03)	0.52 (0.02)
Happy	0.34 (0.01)	0.13 (0.01)	0.14 (0.02)	0.13 (0.01)	0.11 (0.01)	0.57 (0.01)	0.44 (0.03)	0.44 (0.03)	0.55 (0.02)	0.51 (0.02)
Neutral	0.34 (0.01)	0.14 (0.03)	0.14 (0.01)	0.10 (0.01)	0.11 (0.01)	0.57 (0.01)	0.43 (0.03)	0.43 (0.03)	0.5 (0.02)	0.51 (0.03)

RMS contrast was higher and Pixel Intensity lower, for the eyes than for the nose and mouth. PI for mouth fixation was highest for happy faces.

Faces (6.30° horizontally by 10.44° vertically) were presented on a computer screen (75 Hz refresh rate) 70 cm in front of the participants. Fixation was always centered on the screen, but faces were presented offset so that the feature of interest was fixated (i.e. was at the fovea). Four fixation location conditions were hence created: left eye, right eye (middle of the iris for each), nose (tip) and mouth (middle). All trials began with a black fixation cross, which participants needed to fixate on for 307 ms for the trial to begin (Fig. 1). A face then appeared for 257 ms, followed by the response screen. After response, participants were instructed to blink. Each participant completed 80 face trials per condition across 10 blocks of 96 faces (3 emotions × 4 fixations × 8 identities), then completed the anxiety-related STICSA questionnaire. Note the oddball group also had 24 trials where flowers were presented at fixation locations corresponding to those on the face.

For the gender discrimination (GD) task, the response screen was a black question mark which prompted participants to differentiate the gender of the face using index fingers to press buttons on a game controller (counterbalanced across participants). For the emotion discrimination (ED) task, participants selected the emotion of the face from a list on the screen using a mouse. For the oddball (ODD) task, participants pressed the spacebar on a keyboard when an infrequent flower stimulus was presented. A fixation cross was presented in lieu of the response screen for 747 ms on face trials and until a response on flower trials.

#### Electrophysiological and eye-tracking recordings

The EEG was continuously recorded at 516 Hz by an Active-two Biosemi system. The cap contained 66 electrode channels (64 from the extended 10/20 system and two extra posterior electrodes [PO9, PO10]), along with two electrodes around each eye (i.e., on infra-orbital ridges and outer canthi; 4 in total) and two on the mastoids. This system’s ground was the Common Mode Sense (CMS) and Driven Right Leg (DRL) electrodes.

Eye movements were recorded at a sampling rate of 1000 Hz using a remote SR Research Eyelink 1000 eye tracker. The dominant eye was calibrated using a nine-point automated calibration test. This process was redone if a single-point error of 1° was seen, or if the average of all nine-points was greater than 0.5°. During the experiment, a drift correction was used if participants took longer than 10 s to fixate on the fixation cross. A mid-block re-calibration was done after two drift corrections occurred. Participants’ head remained stabilized in a chin rest for the entirety of the experiment.

#### Data processing

Trials with incorrect responses or a saccade of over 1.4° of visual angle away from the desired fixation were rejected. The remaining trials were processed using EEGLab version 13.6.5b^92^ and ERPLab version 5.1.1.0 (http://erpinfor.org/erplab) toolboxes running under Matlab 2014b and Matlab 2018b. The waveforms were set to 500 ms epochs including a 100 ms pre-stimulus baseline (− 100 ms to 400 ms post stimulus onset). A second round of trial rejection occurred, where trials with ± 70 µv artifacts on any electrode were automatically rejected. Finally, visual inspection allowed for the removal of any remaining visible artifacts. Data were filtered with a 0.01–30 Hz bandpass filter. EEGLab .set files were output at this stage and were then used in the MUA described below. As MUA require a minimum of 50 trials per condition for reliability, due to bootstrapping methods^89^, participants with less than 40 trials in any given condition or an overall average of less than 50 trials per condition, were rejected (12 in total). There was an average of 60.48 (6.89 SD) artifact-free trials per condition and per participant in the final sample.

### Statistical analysis

Using LIMO EEG^62^ (EEG-Master version and Hot-Fix version for bug fixes; https://github.com/LIMO-EEG-Toolbox/limo_tools), the epochs were re-set to 0-352 ms to map onto the analysis done in the original study and to align with recent work^57,58^. As done in this work, we decided to not include the baseline to limit the number of unnecessary comparisons being conducted (to preserve power) and because empirical MUA simulations on the effects of baseline duration are still missing. LIMO EEG uses a hierarchical General Linear Model (GLM) where a regression-based analysis is first computed, at each time point and electrode, on the variance between trials of each condition within each participant. This first level analysis ensures individual variance is being accounted for and outputs regression coefficients, which are then used in the group statistical analysis^62^. At this individual subject level, a GLM processed all subjects’ single trials based on the 12 conditions (3 emotions × 4 fixations). Parameter estimates were obtained using Ordinary Least Squares. A neighborhood electrode distance of 0.3759 was used for consistency with previous analyses using the same electrode net^57–59^. The neighborhood matrix was then visually inspected to ensure all electrodes which should be clustered together were correlated.

At the group (second) level, a 3 Task (GD, ED, ODD) × 4 Fixation Location (left eye, right eye, nose, mouth) × 3 Facial Expression (Fearful, Happy, Neutral) mixed model ANOVA was conducted on the parameter estimates from the first level processing stage, with Task as a between-subject factor and Facial Expression and Fixation Location as within-subject factors. Hotelling T-tests are used in rmANOVA to account for the covariance between measures; thus, there is no need to adjust for sphericity. Although a main effect of Task was found (Fig. 2), there were no interactions with Task for any of the other variables of interest. Therefore, we re-ran the analyses without the Task factor to increase power, using a 4 Fixation Location (left eye, right eye, nose, mouth) × 3 Facial Expression (Fearful, Happy, Neutral) within-subjects repeated measures ANOVA. These ANOVAs were run at every time point and electrode using 1000 Bayesian bootstraps and *p* < 0.05. Follow-up *F-*contrasts were run on the whole epoch, using stringent Bonferroni corrected *p-*values to account for the number of comparisons conducted.

Results were corrected for multiple testing using two types of corrections with bootstrapping clustering technique (1000 bootstraps were used as recommended). We used the Cluster Mass spatial–temporal clustering^93^ and the Threshold-Free Cluster Enhancement (TFCE)^94^. These corrections cluster and sum already significant *F*-values that must align with the 1-α portion of a customized null distribution to be deemed significant^60,89,93^. In LIMO, clusters require a minimum of two channels. Simulations have suggested that TFCE might work better than the Cluster Mass correction^89,94^ although this may depend on the study. Thus, we decided to use both but report only the TFCE results in the text (Cluster Mass results are available in supplementary documents).

### Supplementary Information

Supplementary Information.

## Data Availability

Group results are available on the Open Science Framework at this link: OSF|Datasets for: Effects of feature fixation on the processing of facial expressions of emotion—a mass-univariate analysis of scalp ERPs.
