# Supplementary material for: Mass-univariate analysis of scalp ERPs reveals large effects of gaze fixation location during face processing that only weakly interact with face emotional expression
Source: Sci Rep. 2023 Oct 9;13:17022. doi: 10.1038/s41598-023-44355-5 (PMC10562468; doi:10.1038/s41598-023-44355-5)
Supplement: Supplementary file 1 — Supplementary Information. [file 41598_2023_44355_MOESM1_ESM.docx]

**Supplementary information to the paper “Effects of feature fixation on the processing of facial expressions of emotion – a mass-univariate analysis of scalp ERPs”**

**Authors:** Roxane J. Itier^1^* & Amie J. Durston^1^

**Affiliations:** ^1^Department of Psychology**,** University of Waterloo

**Supplementary Figure S1.** Main effects and interaction from the omnibus mixed model ANOVA (3 Tasks x 4 Fixation locations x 3 Facial Expressions), using *α =* 0.05 and **Cluster-Mass correction** (see Fig.2 for TFCE correction). Task was between-subjects and the other factors were within-subjects. **a)** Task effect; **b)** Fixation Location effect; **c)** Facial Expression effect; **d)** Facial Expression by Fixation Location interaction. Time is presented on the X-axis of each plot (from 0-352ms after face onset), and electrodes are presented on the Y-axis. On the right side, a colour bar indicates the *F-*values strength. Note that Task did not interact with any factor.


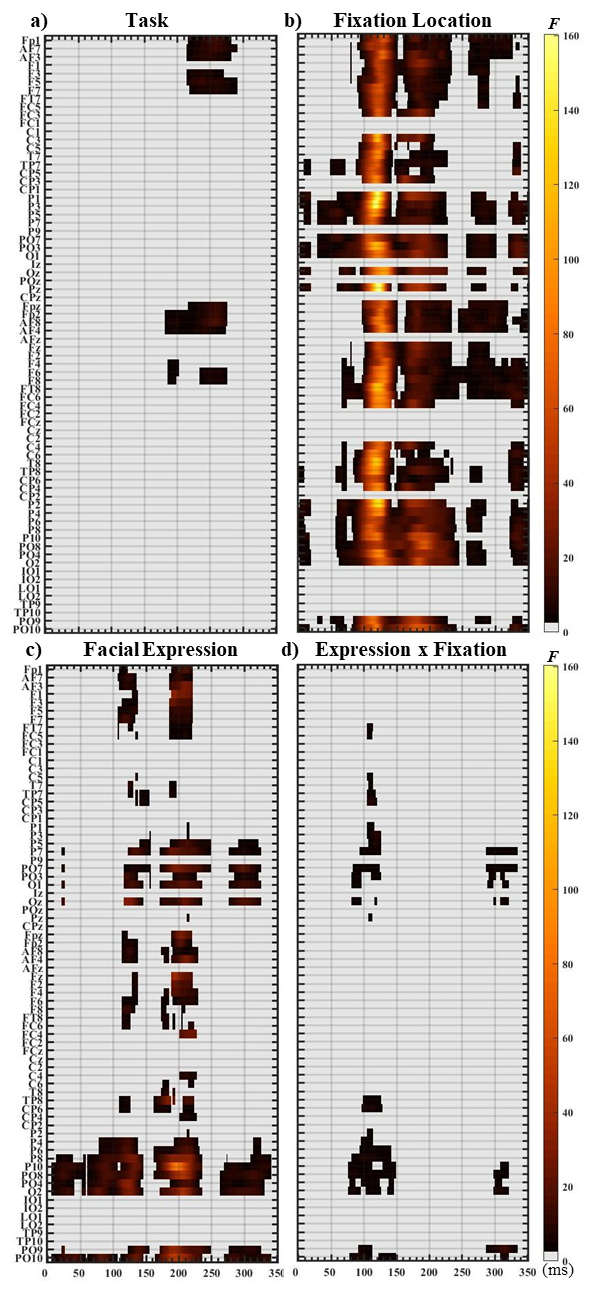


**Supplementary Figure S2.** Main effects and interaction from the omnibus within-subjects repeated measures ANOVA (4 Fixation locations x 3 Facial Expressions). The ANOVA was computed with *α =* 0.05 and used **Cluster-Mass Correction** (see Fig.3, Fig.4 and Fig.5 for TFCE correction). **a)** Fixation Location effect; **b)** Facial Expression effect; **c)** Facial Expression by Fixation Location interaction. Time is presented on the X-axis of each plot (from 0-352ms after face onset), and electrodes are presented on the Y-axis. On the right side of each result, a colour bar indicates the *F-*values strength.

**
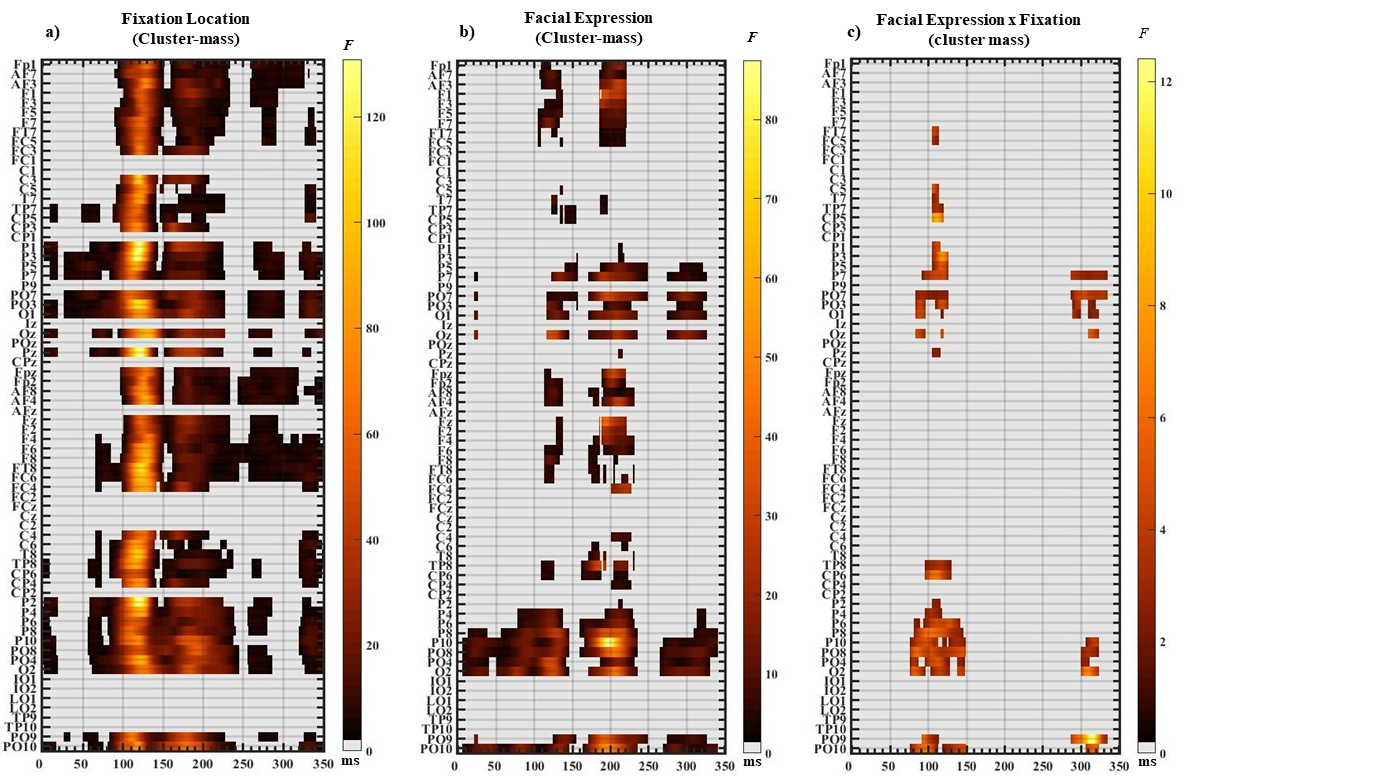
**

**Supplementary Figure S3.** The 6 fixation location contrasts were each computed with α=.008 (to account for the 6 comparisons) and with TFCE corrections applied. The raster plots depict the significant *F*-values at every electrode (y axis) and time-point analyzed (0-352ms post-face onset). *F*-values magnitude are according to the colored scale. Under each raster plot, ERPs for each contrast condition, as well as their difference wave, are displayed at 5 key electrodes: Oz, PO10, PO9, P10, TP8. ERPs and difference waves were computed using inter-subject means and intra-subject 20% trimmed means. Confidence intervals around the difference waves (i.e., Highest Density Interval; HDI) used α=0.008 to align with the analyses. Blue lines at the top of the difference wave plots represent the points that were significant for a given contrast using TFCE correction while the red lines at the bottom of the same plots represent the time points that were significant for that contrast using Cluster-mass correction. From left to right of the figure: **(a)** Left eye (LE) vs Right eye (RE) contrast (with LE minus RE difference wave); **(b)** Left eye (LE) vs. Nose (NO) contrast (with LE minus NO difference wave); **(c)** Left eye (LE) vs. Mouth (MO) contrast (with LE minus MO difference wave); **(d)** Right eye (RE) vs. Nose (NO) contrast (with RE minus NO difference wave); **(e)** Right eye (RE) vs. Mouth (MO) contrast (with RE minus MO difference wave); **(f)** Nose (NO) vs. Mouth (MO) contrast (with NO minus MO difference wave).


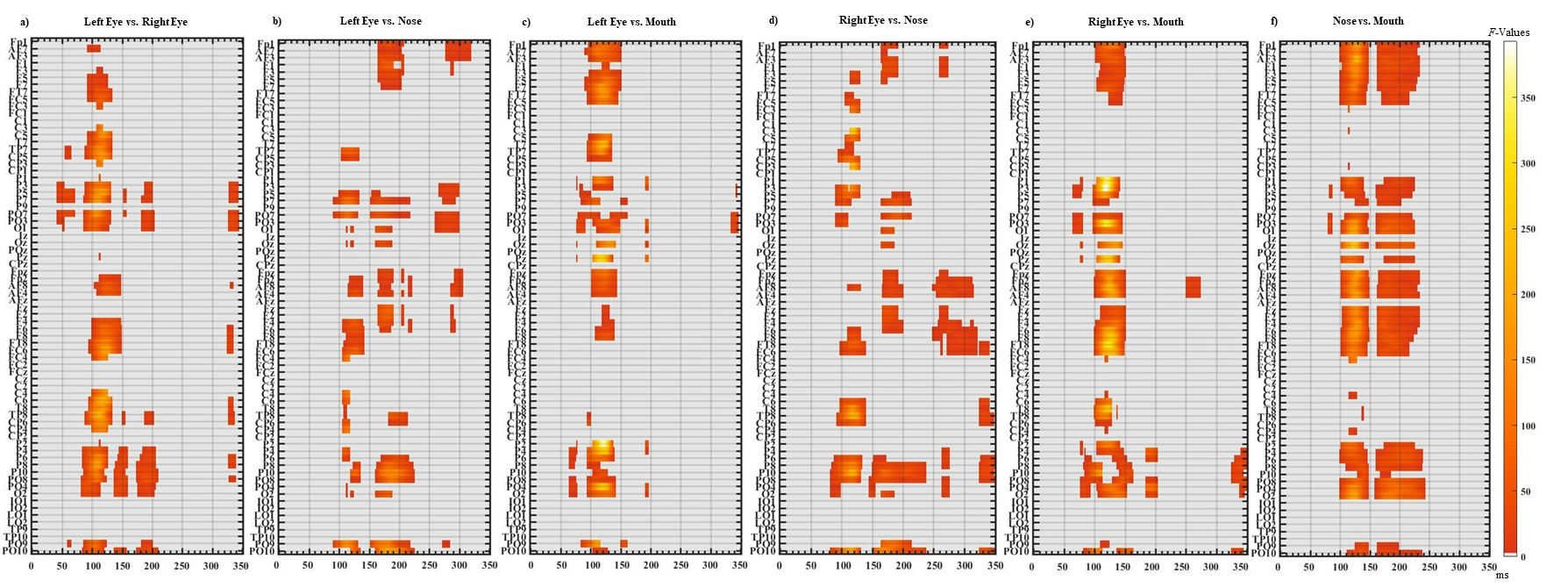


**Supplementary Table 1:** Detailed description of the follow-up paired contrast between fixation locations (Sup.Fig.3 for paired contrasts). The maximum *F* values obtained for each temporal window is reported along with the direction of the effect when applicable, and the amplitude difference (∆µV) between the compared fixation locations.

|  | **Timing** | **Location** | **Maximal F-Value** | **Direction** |
| --- | --- | --- | --- | --- |
| **Left eye vs. Right eye** | 41-70ms | TP7, CP5, P3, P5, P7, P9, PO7, PO3, O1, PO9 | 61ms on PO7 (*F*(1, 53) *=* 42.53, *P* = .001, | *∆µV* = -0.72 (left-eye < right eye), [ -0.17; -1.22] 99.2% CI |
|  | 83-158ms | AF7, F3, F5, F7, FT7, FC5, FC3, C3, C5, T7, TP7, CP5, CP3, P1, P3, P5, P7, PO7, PO3, O1, Pz, Fp2, AF8, AF4, F4, F6, F8, FT8, FC6, FC4, C4, C6, T8, TP8, CP6, CP4, P2, P4, P6, P8, P10, PO8, PO4, O2, PO9, PO10 | TP8 at 111ms (*F*(1, 53) *=* 215.05, *P* = .001, | *∆µV =* -2.06 (left-eye < right eye), [-1.49; -2.67] 99.2% CI; More positive amplitude for the eye fixation ipsilateral to the electrode |
|  | 174-209ms | P3, P5, P7, PO7, PO3, O1, TP8, CP6, P4, P6, P8, P10, PO8, PO4, O2, PO9, PO10 | 192ms on PO8 (*F*(1, 53) *= 51.63, P = .001,* | *∆µV =* -1.13*,* [-0.62; -1.73] 99.2% CI ; Amplitudes were more negative for the ipsilateral eye fixation |
|  | 325-344ms | P3, P5, P7, PO7, PO3, O1, AF8, F6, F8, FT8, FC6, FC4, C6, T8, TP8, CP6, P6, P8, PO8 |  |  |
| **Left eye vs. nose** | 88-142ms | TP7, CP5, P5, P7, PO7, O1, Oz, Fp2, AF8, AF4, F4, F6, F8, FT8, FC6, FC4, C4, C6, T8, TP8, CP6, CP4, P4, P6, P8, P10, PO8, PO4, O2, PO9, PO10 | PO9 at 113ms (*F(1, 53) =* 158.68, *P =* .001) | *∆µV =* 1.89 (Left-eye > Nose), [1.43; 2.52] 99.2% CI |
|  | 152-225ms | Fp1, AF7, AF3, F1, F3, F5, F7, P5, P7, PO7, O1, Oz, Fpz, Fp2, AF8, AF4, Fz, F2, F4, F6, TP8, CP6, P6, P8, P10, PO8, O2, PO9, PO10 | 180ms on PO10 (*F(1, 53) = 188.22, P= .001)* | *∆µV = -1.97 (*Left-eye < Nose*),* [-1.42; -2.45] 99.2% CI |
|  | 260-319ms | Fp1, AF7, AF3, F1, F3, P3, P5, P7, PO7, PO3, O1, Fpz, Fp2, AF8, AF4, Fz, F2, F4, F6, PO9 | 279ms on PO7*(F(1, 53) = 30.87, P = .001)* | *∆µV = 0.61*(Left eye > nose), [0.19; 1.14] 99.2% CI |
| **Left eye vs mouth** | 61-161ms | Fp1, AF7, AF3, F1, F3, F5, F7, FT7, FC5, C5, T7, TP7, CP5, P1, P3, P5, P7, PO7, PO3, O1, Oz, Pz, Fpz, Fp2, AF8, AF4, Fz, F2, F4, F6, F8, TP8, CP6, P2, P4, P6, P8, P10, PO8, PO4, O2, PO9, PO10 | 121ms on P2 (*F(1, 53) =* 376.50, *P =* .001)*,* | *∆µV =* -3.09 (Left-eye<Mouth), [-2.51 ; -3.80] 99.2% CI |
|  | ~190ms | P1, P3, PO3, O1, Oz, Pz, P2, P4, PO4, O2 |  |  |
|  | ~335ms | P3, P5, PO7, PO3, O1 |  |  |
| **Right eye vs Nose** | 80-139ms | F3, F5, FT7, FC5, FC3, C3, C5, T7, TP7, CP5, CP3, P3, P5, P7, PO7, PO3, AF8, F6, F8, FT8, FC6, C6, T8, TP8, CP6, P6, P8, P10, PO8, PO4, O2, PO10 | 119ms on C3 (*F(1, 53)* = 273.08, *P =* .001) | *∆µV = -0.96* (Right-eye < Nose)*,* [-0.69; -1.11] 99.2% CI |
|  | 143-237ms | Fp1, AF7, AF3, F1, F3, F5, P5, P7, PO7, O1, Oz, Fpz, Fp2, AF8, AF4, Fz, F2, F4, F6, P6, P8, P10, PO8, PO4, O2, PO9, PO10 | 166ms on PO10 (*F(1, 53) =* 143.97, *P =* .001) | *∆µV =* -1.33 (Right-eye < Nose), [-0.86; -1.85] 99.2% CI |
|  | 250-321ms | Fp1, AF3, F1, F3, Fpz, Fp2, AF8, AF4, Fz, F2, F4, F6, F8, FT8, FC6, P4, P6, P8, PO8, PO4, O2 | 272ms on AF4 (*F(1, 53) =* 31.89, *P =* .001) | *∆µV* = -0.47 (Right-eye < Nose), [-0.18; -0.84] 99.2% CI |
|  | 323-352ms | FT8, FC6, C6, T8, TP8, CP6, P6, P8, P10, PO8, PO10 | 332ms on PO10 (*F(1, 53) =* 48.21, *P =* .001) | *∆µV =* -0.60 (Right-eye < Nose), [-0.25; -0.97] 99.2% CI |
| **Right eye vs mouth** | 63-162ms | Fp1, AF7, AF3, F1, F3, F5, F7, FT7, FC5, P1, P3, P5, P7, PO7, PO3, O1, Oz, Pz, Fpz, Fp2, AF8, AF4, Fz, F2, F4, F6, F8, FT8, FC6, FC4, C4, C6, T8, TP8, CP6, CP4, P2, P4, P6, P8, P10, PO8, PO4, O2, PO9, PO10 | 119ms on P3 (*F(1, 53) =* 393.02, *P =* .001) | *∆µV =* -2.97 (Right-eye < mouth), [-2.44; -3.59] 99.2% CI |
|  | 182-205ms | P4, P6, PO8, PO4, O2 |  |  |
|  | 249-274ms | Fp2, AF8, AF4 |  |  |
|  | 323-352ms | P4, P6, P8, P10, PO8, PO4, O2, PO10 |  |  |
| **Nose vs mouth** | 98-150ms | Fp1, AF7, AF3, F1, F3, F5, F7, FT7, FC5, FC3, C3, CP3, P1, P3, P5, P7, PO7, PO3, O1, Oz, Pz, Fpz, Fp2, AF8, AF4, Fz, F2, F4, F6, F8, FT8, FC6, FC4, C4, T8, TP8, CP4, P2, P4, P6, P8, P10, PO8, PO4, O2, PO9, PO10 | 123ms on Oz (*F(1, 53) = 256.07, P =* .001) | *∆µV* = -3.98 (Nose < Mouth), [-3.19; -4.88] 99.2% CI |
|  | 156-244ms | Fp1, AF7, AF3, F1, F3, F5, F7, FT7, FC5, P1, P3, P5, P7, PO7, PO3, O1, Oz, Pz, Fpz, Fp2, AF8, AF4, Fz, F2, F4, F6, F8, FT8, FC6, P2, P4, P6, P8, P10, PO8, PO4, O1, PO9, PO10 | 182ms on O2 (*F(1, 53) = 123.99, P = .001)* | *∆µV =* 2.14 (Nose > Mouth), [1.39; 2.97] 99.2% CI |
